# Impact of Hyaluronic Acid and Other Re-Epithelializing Agents in Periodontal Regeneration: A Molecular Perspective

**DOI:** 10.3390/ijms252212347

**Published:** 2024-11-17

**Authors:** Alessandro Polizzi, Ylenia Leanza, Antonio Belmonte, Cristina Grippaudo, Rosalia Leonardi, Gaetano Isola

**Affiliations:** 1 Department of General Surgery and Surgical-Medical Specialties, School of Dentistry, University of Catania, Via S. Sofia 68, 95124 Catania, Italy; 2 Head and Neck Department, Università Cattolica del Sacro Cuore, Fondazione Policlinico Universitario A. Gemelli IRCCS, Largo A. Gemelli 8, 00168 Rome, Italy

**Keywords:** hyaluronic acid, connective cells, gingival tissues, periodontal regeneration, periodontitis

## Abstract

This narrative review delves into the molecular mechanisms of hyaluronic acid (HA) and re-epithelializing agents in the context of periodontal regeneration. Periodontitis, characterized by chronic inflammation and the destruction of tooth-supporting tissues, presents a significant challenge in restorative dentistry. Traditional non-surgical therapies (NSPTs) sometimes fail to fully manage subgingival biofilms and could benefit from adjunctive treatments. HA, with its antibacterial, antifungal, anti-inflammatory, angiogenic, and osteoinductive properties, offers promising therapeutic potential. This review synthesizes the current literature on the bioactive effects of HA and re-epithelializing agents, such as growth factors and biomaterials, in promoting cell migration, proliferation, and extracellular matrix (ECM) synthesis. By modulating signaling pathways like the Wnt/β-catenin, TGF-β, and CD44 interaction pathways, HA enhances wound healing processes and tissue regeneration. Additionally, the role of HA in facilitating cellular crosstalk between epithelial and connective tissues is highlighted, as it impacts the inflammatory response and ECM remodeling. This review also explores the combined use of HA with growth factors and cytokines in wound healing, revealing how these agents interact synergistically to optimize periodontal regeneration. Future perspectives emphasize the need for further clinical trials to evaluate the long-term outcomes of these therapies and their potential integration into periodontal treatment paradigms.

## 1. Introduction

Periodontitis is a chronic inflammatory disease involving dysbiotic plaque biofilms and the progressive destruction of the tooth-supporting structures, primarily characterized by clinical attachment loss (CAL), alveolar bone loss, periodontal pocketing, and gingival bleeding [1]. The periodontium is composed of four mesenchymal tissue components (gingiva, cementum, alveolar bone, and periodontal ligament [PDL]) that provide the tooth with an attachment apparatus to the jawbone capable of withstanding masticatory forces and maintaining homeostasis in the oral cavity. The PDL, highly cellular and vascularized, contains fibroblasts, endothelial cells, and undifferentiated mesenchymal cells essential for periodontal tissue homeostasis, responding to signals by differentiating into cementoblasts, osteoblasts, or fibroblasts [2]. It has been seen that tissue destruction in periodontitis is largely due to cytokines such as IL-1β and TNF-α, which are activated by inflammatory processes caused by oral pathogens, representing an excessive host response [3]. The main objective of periodontal therapy is therefore the treatment of the infection by removing the biofilm, stopping or slowing down further bone loss. These goals can be achieved with the first and second steps of periodontal treatment. The first step consists of patient motivation for and education on oral hygiene and the control of risk factors such as smoking and diabetes, while the second step consists of debridement, i.e., non-surgical subgingival instrumentation [4]. When biofilm is removed from affected roots, gingival epithelial cells repopulate the wound, forming a long-junctional epithelium on the root surface [2]. However, the optimal control of the subgingival biofilm and inflammation can be challenging in areas inaccessible to mechanical treatment, leading to the use of adjunctive chemotherapeutic agents [5]. One of the most used is chlorhexidine (CHX), which has a broad spectrum of activity against all other organisms within the microbial biofilm. However, the prolonged use of CHX leads to several side effects such as the alteration or loss of the sense of taste; dry mouth; and superficial staining of teeth and tongue [6]. Hyaluronic acid (HA) is notable for its antibacterial, antifungal, anti-inflammatory, angiogenic, and osteoinductive effects, enhancing wound healing in periodontal tissues [7]. Therefore, this study aims to deepen our understanding of the molecular mechanisms by which HA and other agents influence periodontal regeneration, focusing on epithelial and connective cell signaling and gene expression to drive healing.

## 2. Materials and Methods

### Inclusion and Exclusion Criteria

In July 2024, an open bibliographic search without timeline restrictions was performed in the major electronic databases, including PubMed, Scopus, Google Scholar, and Web of Science. The present narrative review was developed using articles with the following inclusion criteria: (1) articles written in English, (2) topic related to the molecular aspects of hyaluronic acid and re-epithelizing agents and/or their application in periodontology, (3) design of the study including in vivo and in vitro studies, prospective and retrospective studies, cross-sectional studies, narrative reviews, systematic reviews, and meta-analyses. Articles were excluded if they were (1) not available in English, (2) unrelated to hyaluronic acid or re-epithelizing agents and their properties and/or applications in the field of periodontology, (3) opinion articles or conference reports.

## 3. Molecular Mechanisms of Hyaluronic Acid

### 3.1. Structure and Properties of Hyaluronic Acid

**Figure 1:**
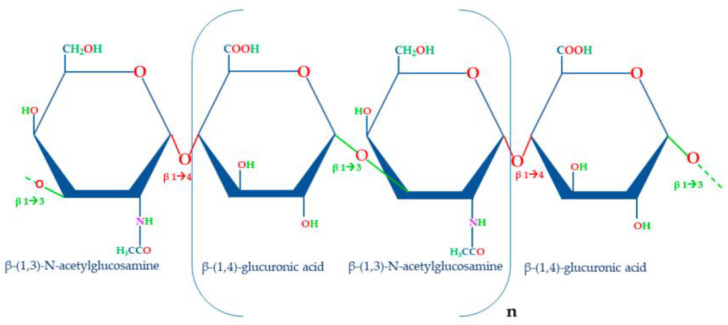
Structure of disaccharide repeating unit of HA. The unit of HA is composed of β-(1,4)-glucuronic acid and β-(1,3)-N-acetylglucosamine linked together by β-1,3 and β-1,4 glycosidic bonds. The molecular weight of this molecule depends on the number of repetitions of the disaccharide unit (n).

Hyaluronic acid (HA) is an important member of the glycosaminoglycans (GAGs). Compared to other GAG members, HA consists of repeating units of (1,4)-glucuronic acid (GA) and (1,3)-N acetylglucosamine (NAG) that are repeatedly linked together by alternating 1,3 and 1,4 glycosidic bonds [8] (Figure 1).

Unlike other glycosaminoglycans which are synthesized within the cell in the Golgi apparatus and then secreted externally by exocytosis, HA is synthesized by three transmembrane enzymes, HA synthetase 1 (HAS1), HA synthetase 2 (HAS2), and HA synthetase 3 (HAS3), on the inner side of the plasma membrane [9]. HAS3 produces molecules with a lower MW (1 105 to 1 106 Da) than that of HAS1 and HAS2 that are able to generate large-sized HA (greater than 2 106 Da) [10]. The equilibrium between HA synthesis and degradation has an important role in determining the amount of the molecule and the MW of HA and, consequently, its properties. In fact, it was proposed that HAs of different molecular sizes can display different and opposing biological activity [11]. For example, high-MW HA exhibits anti-inflammatory activity, controlling the recruitment of inflammatory cells, whereas the low-MW molecules are pro-inflammatory and promote angiogenesis and tissue remodeling in the wound healing process [12].

The presence of carboxylic groups in HA determines a negatively charged molecule with hydrophilic characteristics at physiological pH. As a result, HA has the ability to bind a large amount of water and become viscous through the establishment of intramolecular and intermolecular hydrogen bonds [8].

In addition, other properties of HA are represented by its biocompatibility; it is well tolerated by living tissues and does not cause any adverse reactions or immune responses. HA is non-immunogenic so it does not stimulate an immune response or cause inflammation when injected into the body [13].

### 3.2. Interaction of Hyaluronic Acid with Epithelial and Connective Tissue Cells

HA has many structural and physiological functions within tissues, such as extracellular and cellular interactions, growth factor interactions, regulation of osmotic pressure, and tissue lubrication. In this way, it helps maintain the structural and homeostatic integrity of tissues [14]. It is responsible for forming associations with collagen, fibrin, and other matrix molecules. The early response to tissue injury includes the formation of a temporary matrix rich in hyaluronan and fibrin, which supports the influx of fibroblasts and endothelial cells into the wound site and the subsequent formation of granulation tissue [15]. Whether HA is bound to cells or to extracellular matrix components, its hydrophilic nature creates an environment permissive for the migration of cells to new tissue sites, while its free-radical scavenging and protein exclusion properties offer protection to cells and extracellular matrix molecules against free radical and proteolytic damage. These represent some of the properties that can mediate the healing of both acute and chronic wounds.

HA modulates inflammation by increasing the infiltration of inflammatory cells and extracellular matrix into the wound and stimulating the production of pro-inflammatory cytokines. It also takes part in the organization and stabilization of granulation tissue matrices and scavenges reactive oxygen species, such as superoxide radical (·O_2_) and hydroxyl radical (·OH), thus preventing periodontal destruction [16].

Among its other functions, HA promotes cell migration, proliferation, and differentiation by making the clot more receptive to colonization by cells responsible for regenerating damaged tissue, such as mesenchymal and basal keratinocytes [17]. HA also possesses osteoconductive potential by accelerating bone regeneration by means of the chemotaxis, proliferation, and successive differentiation of mesenchymal cells. HA shares its bone induction characteristics with osteogenic substances such as bone morphogenetic protein-2 (BMP-2) and osteopontin [18]. Finally, HA can act as a biomaterial scaffold for other molecules, such as BMP-2 and platelet-derived growth factor (PDGF), used in guided bone regeneration techniques and tissue engineering research [19].

### 3.3. Role of Hyaluronic Acid in Cell Migration, Proliferation, and Differentiation

**Figure 2:**
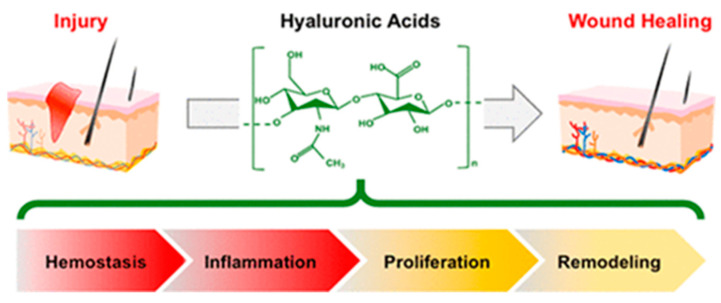
Wound healing process. It comprises hemostasis, inflammation, proliferation, and remodeling. Reprinted with permission from [20]. Copyright 2024 American Chemical Society.

HA plays an important role in cell differentiation, proliferation, and migration throughout tissue development and regeneration [20] (Figure 2).

Proliferation consists of three stages: re-epithelialization (the closure of a wound’s surface), angiogenesis, and the formation of granulation tissue [21]. During cell migration, it binds to CD44 and a receptor for HA-mediated motility (RAHMM) to mediate the production of transforming growth factor β (TGFβ), epidermal growth factor (EGF), basic fibroblast growth factor (bFGF), and vascular endothelial growth factor (VEGF), resulting in the recruitment, maturation, and activation of keratinocytes, endothelial cells [22], and fibroblasts [23]. For example, HA stimulates endothelial cells and fibroblasts to synthesize and depose type III collagen within the wound, resulting in the formation of a new collagen matrix [24]. HA, being an essential extracellular matrix (ECM) component, can promote cellular differentiation. At the final phase of the healing process, HA interacts with CD44 and RAHMM to produce matrix metalloproteinases (MMPs) and TGF-β, which promote the differentiation of fibroblasts into myofibroblasts, facilitating the maturation of collagens (type I) for ECM remodeling [20].

### 3.4. Signaling Pathways Modulated by Hyaluronic Acid in Periodontal Regeneration

The main signaling pathways modulated by hyaluronic acid in periodontal regeneration are as follows:

- CD44, which functions as a receptor for collagen, fibronectin, osteopontin, and HA present in the ECM. The interactions between CD44 and these ECM components transduce several signaling pathways. The cytoplasmic domain of CD44 is bound to the actin cytoskeleton and interacts with cytoskeleton-associated proteins such as ezrin/radixin/moesin (ERM) and ankyrin [25], resulting in cytoskeleton activation and causing several biological functions including cell adhesion, proliferation, and migration [26]. The stimulation of CD44 triggers a signaling cascade associated with two tyrosine kinases: p185 human epidermal growth factor receptor 2 (HER2) and c-Src kinase. The activation of p185HER2 leads to increased cell growth, whereas c-Src kinase activity is responsible for the phosphorylation of cytoskeleton proteins and the induction of cell motility. In addition to the phosphoinositide-3 kinase/phosphoinositide-dependent kinase 1/protein kinase B pathway, Ras protein signaling pathways are also involved in CD44 cytoplasmic signaling [27].
- Other cell surface receptors including RHAMM (Receptor for Hyaluronan-Mediated Motility), LYVE-1 (Lymphatic Vessel Endothelial Hyaluronan Receptor 1), and Layilin, are known to bind to HA. RHAMM is a protein involved in the regulation of cell migration and proliferation, mainly through interactions with hyaluronic acid. It is associated with processes such as wound healing and tumor progression, contributing to the growth and spread of cancer cells [28]. Several kinases, such as Src kinase, focal adhesion kinase, extracellular-signal-regulated kinases (ERK) 1/2, and protein kinase C, are involved in RHAMM signaling. This receptor is also associated with the Ras protein and the Ras signaling pathway [27]. LYVE-1 is a receptor present in the endothelial cells of lymphatic vessels and is involved in the transport of hyaluronic acid. It plays a key role in the regulation of lymphatic drainage and the migration of immune cells. Layilin is a membrane protein that regulates the interaction of cells with their surroundings, influencing processes such as cell migration and adhesion. The HA-induced regulation of cellular function via HA receptors varies depending on differences in the cell type, cell origin, and HA size [28].

## 4. Molecular Mechanisms of Re-Epithelizing Agents

Periodontal re-epithelializing agents are substances that help promote the regeneration and healing of periodontal tissues. The main re-epithelializing agents include growth factors, enamel matrix proteins, blood-derived products, engineered tissues, stem cells, biomaterials, and antimicrobial and anti-inflammatory agents.

### 4.1. Growth Factors

The main cellular events in tissue repair, such as mitogenesis, migration, and metabolism, are coordinated by growth factors. The growth factors that may contribute to periodontal regeneration include platelet-derived growth factor, insulin-like growth factor, transforming growth factor-beta, and bone morphogenetic proteins [29].

- Platelet-derived growth factor (PDGF): PDGF is regarded as one of the principal wound-healing hormones. It was discovered by Lynch et al. [30] to promote the regeneration of bone, cementum, and periodontal ligament in the late 1980s. PDGF is composed of two disulfide-bonded polypeptide chains that are encoded by two different genes, namely, PDGF-A and PDGF-B. In nature, PDGF can exist as a homodimer—PDGF-AA and PDGF-BB. PDGF is an important stimulator of cell chemotaxis, proliferation, and matrix synthesis that exhibits anti-apoptosis activity. PDGF is involved in almost all types of wound healing by virtue of platelets and its dual role as a reservoir of growth factors and a hemostasis factor. PDGF stimulates the influx of neutrophils to the wound site [31].
- Insulin-like growth factor: IGFs constitute a family of single-chain proteins that share 49% homology with pro-insulin. Two well-described members of this group are IGF-1 and IGF-2, which are similar in structure and function but are independently regulated. The IGF family includes three ligands and three cell surface receptors, namely, insulin, IGF-I, and IGF-II and Insulin, IGF-1, and IGF–l-mannose G-phosphate receptors, respectively. They have at least six high-affinity IGF-binding proteins which bind circulating IGFs and modulate their biological activity. Both IGF-I and IGF-II are synthesized as large precursor molecules (195 and 156 aa), which are proteolytically cleaved to release the biologically active monomeric proteins. Insulin-like growth factor-I is found in substantial levels in platelets and is released during clotting along with the other growth factors present in platelets. IGF-I released from platelets or produced by fibroblasts may promote the migration of vascular endothelial cells into the wound area, resulting in increased neovascularization. It also stimulates the mitosis of many cells in vitro such as fibroblasts, osteocytes, and chondrocytes [32].
- Transforming growth factors (TGFs): TGFs are a family of structurally and functionally unrelated proteins that have been isolated from normal and neoplastic tissues. The two main polypeptides are TGF-α and TGF-β. TGF-α is a polypeptide sharing 80% homology with epidermal growth factor (EGF) and it binds to the cellular EGF receptor. TGF-β acts as a progression factor for fibroblasts. TGF-β appears to be a major regulator of cell replication and differentiation. It is bifunctional or pleiotropic and can therefore stimulate or inhibit cell growth. TGF-β can also modulate other growth factors such as PDGF, TGF-α, EGF, and fibroblast growth factor (FGF), possibly by altering their cellular response or by inducing their expression [33]. It has a potent effect on matrix synthesis, giving rise to the increased production of collagen and fibronectin and the decreased production of matrix-degrading enzymes [31].
- BMPs (bone morphogenic proteins): BMPs are a group of osteoinductive proteins obtained from nonmineralized bone matrix; they can stimulate the differentiation of pluripotent mesenchymal cells to osteoprogenitor cells. Currently, there are more than 30 identified BMPs, but the most recent studies on periodontal regeneration have been conducted using GDF-5 and BMP-7. They are responsible for several biological activities involving tissue morphogenesis, regeneration, healing, and cell differentiation processes [34]. GDF-5 induces chondrogenesis and osteogenesis in vitro and in vivo, supported by a carrier agent. The bioresorbable polymer polylactic-glycolic acid (PLGA) and specific additives, designed as carriers for rhGDF-5 in minimally invasive regenerative procedures, create an ideal matrix to enhance natural wound healing and the action of rhGDF-5 [35]. BMP-7 is a potent bone-inducing factor and was shown to promote periodontal regeneration in vivo and in vitro. BMP-7 treatment markedly stimulated cementoblast-mediated biomineralization in vitro compared to untreated cells [36].

### 4.2. Enamel Matrix Proteins (EMDs)

EMDs are a heterogeneous mixture of mainly amelogenin-derived proteins produced during tooth development [37]. The major (>95%) constituent of EMDs is amelogenins, a family of hydrophobic proteins derived from a single gene by alternative splicing and controlled post-secretory processing [38]. The amelogenins self-assemble into nanospheres that bind to hydroxyapatite crystallites, organizing the enamel matrix and modulating crystal growth [39]. The hydrophilic carboxy-terminal region of amelogenin is cleaved during binding to apatite. As enamel formation progresses, sequential cleavages of the carboxy-terminal parts modulate apatite binding, making the polypeptides soluble, which are then absorbed by ameloblasts. Amelogenin nanospheres are broken down, and once the full enamel thickness is formed, the protein matrix is replaced by tissue fluid, allowing enamel crystals to grow and fill the space. EMDs have also been shown to increase the autocrine synthesis of TGF-β [40]. This study also reported that EMDs stimulate the autocrine production of other growth factors such as vascular endothelial growth factor (VEGF), PDGF, and cytokines such as interleukin (IL)-6. Together, these observations suggest that specific amelogenin molecules can trigger an appropriately balanced and sequenced autocrine release of growth factors that orchestrate the regenerative effects of EMDs [38].

### 4.3. Blood-Derived Products

These are platelet-rich plasma (PRP), platelet-rich-fibrin (PRF), BMPs, platelet-derived growth factor (PDGF), parathyroid hormone (PTH), and EMDs [41]. They allow for the formation of a fibrin network while combining stem cells, leukocytes, platelets, and cytokines [40]. Furthermore, the platelets in this mix are able to release platelet-derived growth factor, enhancing the osteogenic potential and therefore improving the healing of the lesion [41]. Blood-derived products are relatively easy to acquire, as they only need the patient’s blood sample, which is processed in centrifugal equipment for the separation of blood products. Therefore, the associated risks with this procedure are minimal [40].

### 4.4. Engineered Tissues

Tissue engineering has emerged as an alternative approach to alleviate the shortcomings of conventional therapeutic options by regenerating living and functional dental structures [42]. Tissue engineering draws on the principles of cell biology, developmental biology, and biomaterials science to fabricate new structures to replace lost or damaged tissues [43].

**Figure 3:**
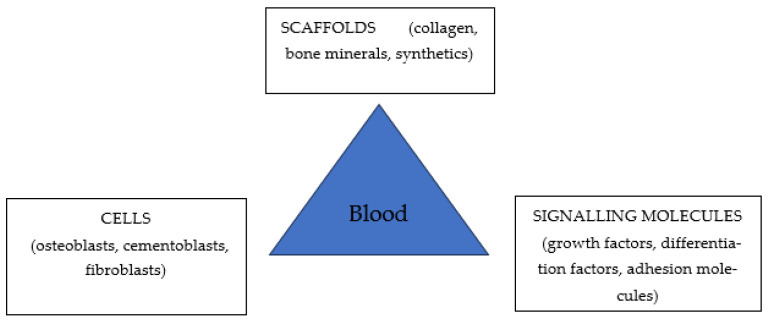
Schematic representation of periodontal tissue engineering.

A successful outcome of periodontal tissue engineering (Figure 3) requires an adequate supply of appropriate progenitor cells with the capacity to differentiate into the required mature tissue-forming phenotypes, including osteoblasts, cementoblasts, and fibroblasts; the appropriate signaling molecules to modulate cellular differentiation; and a conductive three-dimensional extracellular matrix scaffold to support and facilitate these processes. Angiogenic signals, promoting new vascular networks, are essential to provide the nutritional base for tissue growth and homeostasis [44]. Scaffolds must support cell colonization, migration, growth, and differentiation, along with appropriate physicochemical properties, morphology, and degradation kinetics. They should stimulate tissue growth, remodeling, and maturation, providing rigidity and geometry to compensate for the mechanical function of compromised tissue.

### 4.5. Stem Cell

Mesenchymal stem cells (MSCs) are adult stem cells able to give rise to multiple specialized cell types. Due to their extensive distribution in many adult tissues, including those of dental origin, MSCs have become an attractive target for use in periodontal regeneration [45]. Current stem cell-based therapies in periodontology are mainly based on the administration of culture-expanded cells to the periodontal defect to enhance wound healing, and this method is simple and minimally invasive [46].

### 4.6. Biomaterials

Biomaterials play a pivotal role in preventing undesired epithelial and gingival fibroblast cell migration as well as guiding other periodontal tissue regeneration. The most used biomaterials are barrier membranes and bone grafts, used with guided tissue regeneration and/or guided bone regeneration (GTR/GBR) approaches.

- Barrier membranes: GTR/GBR membranes prevent unwanted cell invasion and maintain mechanical stability to support periodontal tissue regeneration. They are categorized into resorbable and non-resorbable membranes. The most common resorbable membranes are made of collagen and are degraded enzymatically by collagenases, polymorphonuclear/macrophage leukocyte-derived enzymes, and bacterial proteases [47]. Non-absorbable barrier membranes are made of expanded polytetrafluoroethylene (ePTFE), a material that exhibits excellent biocompatibility and mechanical stability [48]. Since PTFE is a non-absorbable membrane, patients require a second surgery to retrieve it, which increases the risk of site morbidity [49].
- Bone graft: Bone grafts are commonly used with barrier membranes to obtain periodontal regeneration and the reconstruction of the alveolar ridge. They can be divided into autografts, allografts, xenografts, and alloplastic materials [50]. Autografts are taken from the patient’s body and are considered the gold standard. Allografts are harvested from one individual for transplantation to another [51]. Xenografts are obtained from different species and prepared by various procedures. Two of the most used xenografts in dentistry are deproteinated bovine bone matrix (DBBM) and demineralized porcine bone matrix (DPBM) [52]. Alloplastic grafts are synthetic biomaterials and the most commonly used ones are HA, tricalcium phosphates (TCPs), and bioactive glasses [51].

### 4.7. Antimicrobial Agents

There are different options of antimicrobials that can be locally applied into mucosa like chlorhexidine (CHX), metronidazole, minocycline hydrochloride (MH), doxycycline, and tetracycline.

- CHX: CHX has been shown to be an effective agent against oral biofilms and has antimicrobial properties against both Gram-positive and Gram-negative bacteria, yeasts, and viruses [53]. The mechanism of action (MOA) of CHX begins with the rapid attraction of the cationic CHX molecule to the surface of the bacterial cell, which is negatively charged and contains phosphate and sulfate groups [6]. This causes specific and strong adsorption to the phosphate-containing components that form the surface of the bacterial cell. Penetration through the bacterial cell wall occurs by passive diffusion, damaging the cytoplasmic membrane [54]. This process allows CHX to penetrate the inner cell membrane, increasing permeability. As a result, there is leakage of low-molecular-weight molecules and cytoplasmic components from the microorganism, such as potassium ions, which leads to the inhibition of the activity of some enzymes associated with the cytoplasmic membrane [55]. At this point, the antimicrobial action of CHX remains in the bacteriostatic phase but can be reversed if CHX is removed. If the concentration of CHX remains stable over time or increases, irreversible cellular damage occurs, activating a bactericidal effect. In the bactericidal phase, coagulation and cytoplasmic precipitation occur through the formation of complexes with phosphorylated compounds, such as adenosine triphosphate and nucleic acids [54]. Due to the negative charge of most oral surfaces, such as mucous membranes, teeth, and salivary glycoproteins, cationic CHX molecules adhere well to these surfaces. This interferes with bacterial adhesion and ensures substantivity for up to 12 h [55,56]. Several studies have shown that CHX mouthwash with concentrations between 0.1% and 0.2% has significant anti-inflammatory and anti-plaque effects on the gingiva and teeth [57,58,59]. However, CHX has adverse effects such as xerostomia, hypogeusia, and discoloration of the tongue, tartar, and tooth staining with long-term use. Less common adverse effects include swelling of the parotid gland, oral paresthesia, glossodynia, and desquamation of the oral mucosa [60].
- Metronidazole: Metronidazole is a synthetic antibiotic derived from azomycin and it is very effective in treating infections caused by anaerobic or microaerophilic microorganisms [61]. The mechanism of action of metronidazole consists of crossing the cell membrane of the target microorganism by passive diffusion. Once inside the cell, the nitro group of metronidazole is reduced to nitro radicals by ferredoxin or flavodoxin. Its selectivity for anaerobic bacteria and microaerophilic microorganisms arises from the redox potential of their electron transport systems, which are responsible for the reduction of nitro groups and the generation of toxic metabolites. These metabolites, such as N-(2-hydroxyethyl) oxamic acid and acetamide, can interact with DNA and form covalent bonds with guanosine, thereby impairing DNA replication and function [62]. Due to the low rate of bacterial resistance to metronidazole [63] and its spectrum of action against Gram-negative bacteria associated with periodontal diseases, this appears to be a promising drug for the treatment of periodontitis [64].
- Minocycline, doxycycline, and tetracycline: These substances are greatly effective in the inhibition of Gram-negative facultative anaerobes [65] and exhibit anti-collagenase activity [66]. Tetracyclines are bacteriostatic antibiotics that exert their antibacterial activity by inhibiting microbial protein synthesis. Doxycycline and minocycline are more lipophilic than tetracycline, allowing them to pass directly through the bacterial cell membrane’s lipid bilayer. Once across this layer, an energy-dependent mechanism transports the drug through the inner cytoplasmic membrane. Inside the cell, tetracycline specifically binds to the 30S ribosomal subunit. This binding appears to prevent the aminoacyl-tRNA from attaching to the mRNA ribosomal receptor site, thereby blocking the addition of the amino group to the growing peptide chain [66]. Minocycline is better for its effect on adhesion and diffusion of fibroblasts, crucial for tissue regeneration [67]. Doxycycline has a greater protein-binding capacity, a longer half-life, and is the most potent tetracycline for collagenase inhibition [66].

### 4.8. Anti-Inflammatory Agents

**Figure 4:**
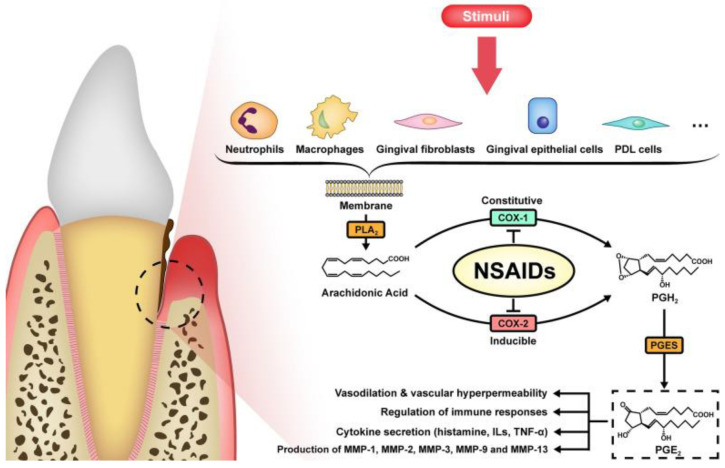
The synthesis and effects of PGE2 in periodontitis. From [72], under Creative Commons Attribution 4.0 International License.

The disease severity largely depends on the immune response, which drives periodontal tissue destruction and influences treatment efficacy [68]. Therefore, various host modulators have been suggested to complement traditional therapies. Among these, nonsteroidal anti-inflammatory drugs (NSAIDs) are the most well known and frequently used [69]. They work by blocking cyclooxygenase (COX), which exists in the form of two isoforms. COX-1 is the constitutive isoform responsible for basal prostaglandin production, which maintains physiological functions such as platelet activation and gastrointestinal mucosal protection. In contrast, COX-2 is inducible, expressed in response to inflammatory stimuli (such as hypoxia, IL-1, IFN-γ, and TNF-α), and produces prostaglandins that promote inflammation, swelling, and pain at the inflammation site [70]. Both isoforms catalyze the conversion of arachidonic acid into prostaglandin G2 (PGG2). PGG2 is then reduced to prostaglandin H2 (PGH2) via a subsequent peroxidase reaction. PGH2 serves as a precursor for the synthesis of various biologically active primary prostaglandins, including PGD2, PGE2, PGF2α, prostacyclin (PGI2), and thromboxane A2 (TxA2) [71]. NSAIDs inhibit PG synthesis, exerting analgesic, antipyretic, and anti-inflammatory effects [72] (Figure 4).

## 5. Crosstalk Between Epithelial and Connective Tissue Cells

### 5.1. Interactions Between Epithelial and Connective Tissue Cells in Periodontal Tissues

The interaction between epithelial and connective cells is multifactorial, involving a wide variety of cytokines, growth factors, and cell adhesion molecules. These factors regulate processes such as cell proliferation, migration, and differentiation, influencing both the health of periodontal tissue and the progression of periodontal diseases [73]. In particular, fibroblasts within the connective tissue, where they play a crucial role in the synthesis of extracellular matrix (ECM) components and in matrix remodeling, interact with epithelial cells through cell–cell contact and soluble mediators, thus influencing cell proliferation, differentiation, and migration [74]. Epithelial cells and fibroblasts therefore engage in bidirectional communication to maintain tissue integrity and respond to external stimuli. Epithelial cells secrete growth factors such as epidermal growth factor (EGF) and transforming growth factor-beta (TGF-β), which modulate fibroblast activity, promoting collagen synthesis and ECM deposition [75]. In contrast, fibroblasts produce signaling molecules such as keratinocyte growth factor (KGF) and hepatocyte growth factor (HGF), which stimulate epithelial cell proliferation and migration. This bidirectional communication is the basis of wound healing and the maintenance of a healthy periodontal environment [76]. Epithelial cells produce pro-inflammatory cytokines, such as interleukin-1 (IL-1), IL-6, and tumor necrosis factor-alpha (TNF-α), in response to pathogenic stimuli. These cytokines activate fibroblasts in the underlying connective tissue, promoting the production of matrix metalloproteinases (MMPs), enzymes that degrade the extracellular matrix during the inflammatory response and tissue remodeling [3]. For cells, the ECM provides a structural scaffold and plays a role in determining their behavior through mechanical and biochemical signals. ECM components, including collagen, elastin, fibronectin, and laminin, interact with integrins, influencing cell adhesion, migration, and differentiation. ECM remodeling is a process based on the balance between its synthesis and degradation. Matrix metalloproteinases (MMPs), particularly MMP-1, MMP-8, and MMP-13, are produced by both epithelial and connective tissue cells and are crucial for ECM turnover. The dysregulation of MMP activity occurs with the progression of periodontal disease in which we see excessive degradation of collagen and other matrix components [77]. In periodontal diseases, such as gingivitis and periodontitis, the interactions between epithelial and connective tissue cells are altered by the infiltration of pathogens and the resulting immune response. Pathogens, such as Porphyromonas gingivalis and Aggregatibacter actinomycetemcomitans, cause the activation of epithelial cells and the production of pro-inflammatory cytokines that stimulate fibroblasts and other immune cells, resulting in an inflammatory response and degradation of periodontal tissues [78]. MMPs produced by fibroblasts in this process degrade the extracellular matrix and collagen that constitute the supporting structure of the periodontal ligament. Epithelial cells also act as sentinel cells of the innate immune response, recognizing pathogens via toll-like receptors (TLRs) and activating a signaling cascade that promotes inflammation. Fibroblasts, on the other hand, are involved in adaptive immunity, interacting with T and B lymphocytes, modulating their function through the production of cytokines such as IL-10 and TGF-β, which have immunoregulatory effects [79]. Periodontal ligament fibroblasts and mesenchymal stem cells (MSCs) are important in periodontal tissue regeneration. Interactions between MSCs and epithelial cells are mediated by fibroblast growth factor (FGF), insulin-like growth factor (IGF), and signals derived from the extracellular matrix. These signals promote cell proliferation, migration, and differentiation, facilitating periodontal tissue regeneration [80].

### 5.2. Impact of Hyaluronic Acid and Re-Epithelizing Agents on Epithelial–Connective Tissue Crosstalk

Hyaluronic acid and various re-epithelializing agents, such as cytokines and growth factors, have been shown to significantly influence epithelial–connective tissue interactions. HA acts on various cell types, including fibroblasts, keratinocytes, and immune cells, to support tissue repair. It binds to receptors like CD44, RHAMM, and ICAM-1, modulating cell migration, proliferation, and ECM synthesis [81]. Re-epithelializing agents, such as growth factors like EGF (epidermal growth factor), TGF-β (transforming growth factor-beta), and cytokines such as IL-6 (Interleukin-6) and TNF-α (tumor necrosis factor-alpha), play an important role in modulating epithelial–connective tissue interactions [82]. HA can influence these factors by facilitating their release and interaction with cell receptors, impacting inflammation and repair balance [83]. The interaction between hyaluronic acid and re-epithelializing agents is diverse. For example, HA can modulate the activity of EGF and TGF-β. It can interact with TLR4 (Toll-like receptor 4), affecting the balance between inflammation and repair during tissue regeneration. HA also modulates the proliferation of keratinocytes and fibroblasts, which are essential for the wound healing process. The binding of HA to CD44, a receptor on the surface of keratinocytes, stimulates intracellular signaling pathways, such as the ERK1/2 (Extracellular Signal-Regulated Kinase) and PI3K/Akt (Phosphoinositide 3-kinase/Protein kinase B) pathways, which promote cell proliferation and migration. Instead, in fibroblasts, HA stimulates the production of collagen [84]. Epithelializing agents, such as epidermal growth factor (EGF), transforming growth factor-beta (TGF-β), and inflammatory cytokines (e.g., IL-6, TNF-α), act synergistically with hyaluronic acid to modulate the interaction between epithelial and connective cells. EGF is responsible for the proliferation and migration of keratinocytes. When EGF binds to EGFR, it activates an intracellular signaling cascade that promotes cell proliferation and ECM synthesis, thereby accelerating wound closure [85]. TGF-β is essential in the wound maturation phase as it regulates the transition of fibroblasts into myofibroblasts, a phase essential for wound contraction and the synthesis of new connective tissue. HA modulates this transition by interacting with TGF-β receptors on the cell surface. Cytokines such as IL-6 and TNF-α are released by immune cells and play a key role in the inflammatory phase of wound healing. Low-molecular-weight HA can activate Toll-like receptors (TLR2 and TLR4) on immune cells, modulating the production of these cytokines [86]. The combined action of HA and re-epithelializing agents optimizes the wound healing response. For example, studies in murine models have shown that the application of HA in combination with EGF can significantly accelerate re-epithelialization and reduce scar formation, suggesting a synergistic effect between the two [87]. HA also plays an important role in modifying the extracellular matrix (ECM) during wound healing. The interaction of HA with ECM proteins, such as fibronectin and collagen, can influence its structure and function, enhancing cell adhesion, migration, and proliferation [27]. At the molecular level, the interaction between HA and re-epithelializing agents modulates several cellular signaling pathways, including the MAPK (Mitogen-Activated Protein Kinase), Akt, and NF-κB (Nuclear Factor kappa-light-chain-enhancer of activated B cells) pathways. These pathways are critical for regulating the inflammatory response, cell proliferation, and ECM synthesis [82].

### 5.3. Effects of Crosstalk on Periodontal Regeneration Processes

The main crosstalk mechanism regarding periodontal regeneration is the interaction between immune cells (such as macrophages and T lymphocytes) and mesenchymal stem cells (MSCs) present in the periodontal ligament (PDL). MSCs could differentiate into different cell lineages, including osteoblasts and fibroblasts, which are essential for periodontal tissue regeneration. Immune cells release cytokines and growth factors that influence MSC behavior. For example, IL-10 produced by M2-type macrophages has been shown to promote MSC proliferation and differentiation into osteoblasts, favoring bone regeneration [88]. In contrast, IFN-γ and IL-17, released by activated T lymphocytes, can determine a pro-inflammatory phenotype in MSCs, inhibiting their regenerative capacity [89]. Bone regeneration requires a balance between bone formation and bone resorption, mediated by osteoblasts and osteoclasts, respectively. Crosstalk between these two cell types is essential to control bone remodeling during periodontal regeneration. Osteoprotegerin (OPG) and RANK ligand (RANKL) are key factors modulating this interaction. Osteoblasts release OPG, which acts as a RANKL inhibitor by binding its receptor on osteoclast precursors, thus preventing their differentiation into mature osteoclasts and, consequently, limiting bone resorption. Under inflammatory conditions, the RANKL levels increase, promoting osteoclast activity and impairing bone regeneration [90]. The crosstalk also involves several molecular signaling pathways that regulate the cellular response during periodontal regeneration. Among these, the Wnt/β-catenin and Notch pathways play key roles. The Wnt/β-catenin pathway is known to promote osteoblast differentiation and bone formation [91]. However, this pathway can be negatively modulated by inflammatory signals, such as TNF-α, which reduce β-catenin activity, hindering bone regeneration [92]. Instead, the Notch signaling pathway is involved in the regulation of stem cell differentiation and the balance between bone formation and resorption. In particular, the activation of Notch signaling has been shown to inhibit osteoblast differentiation, demonstrating an important role in the negative regulation of bone regeneration [93]. Appropriate crosstalk between cells and molecular factors can promote periodontal regeneration processes. For example, communication between M2 macrophages and MSCs, through the production of IL-10 and TGF-β, can promote an anti-inflammatory environment and promote osteoblastic differentiation and bone formation [94]. In addition, the collaboration between growth factors such as FGF-2 (fibroblast growth factor 2) and PDGF (platelet-derived growth factor) can stimulate periodontal ligament fibroblast proliferation and extracellular matrix synthesis, thus accelerating connective tissue regeneration [95]. Crosstalk can also have negative effects on regenerative processes, especially under conditions of chronic inflammation. Pro-inflammatory cytokines such as TNF-α and IL-1β, released by activated immune cells, can impair MSC function by inhibiting their proliferation and differentiation [96]. An imbalance in osteoblast–osteoclast crosstalk, due to altered levels of RANKL and OPG, can lead to excessive bone resorption and failure of periodontal regeneration [90].

### 5.4. Molecular Mechanisms Underlying Epithelial–Connective Tissue Interactions in Periodontal Regeneration

Epithelial cells express cell adhesion molecules, such as cadherins and integrins, which mediate cell adhesion and communication between epithelial cells and cells of the underlying connective tissue.

Molecules involved in epithelial–connective tissue interactions are as follows:

- Integrins and cadherins: Integrins such as α6β4 and αvβ6 are very relevant in connective tissue–epithelium interactions, as they influence cell migration and proliferation during periodontal regeneration. Cadherins, on the other hand, are cell–cell adhesion molecules that stabilize epithelial junctions and modulate epithelial–connective tissue interactions.
- Cytokines and growth factors: Several growth factors, such as EGF, TGF-β, and PDGF, are involved in epithelial–connective tissue interactions. These factors modulate cell proliferation, differentiation, and matrix production, contributing to tissue regeneration [97].

## 6. Clinical Implications and Future Perspectives

### 6.1. Clinical Implications

Several studies have shown that the mechanical removal of plaque, carried out by the patient, is not sufficient for healing [98,99,100]. Also, the presence of periodontal pockets, their depth, and anatomical factors, such as furcations in the molars, limit the effectiveness of debridement in NSPT [101]. Therefore, we will see specifically how re-epithelizing agents can be used as topical adjuvants in NSPT and their effect on the periodontium.

- HA: Its numerous properties have been studied as a possible adjuvant in NSPT. Its application has been tested in various periodontal conditions [102,103,104], including the treatment of infraosseous defects, where a reduction in PD and an increase in CAL were recorded [105]. Among the most important properties is its regenerative capacity, linked to its osteoinductive effect, which promotes the migration of endothelial cells that form a network for the deposition of bone tissue [106].
- Growth factors: Plasma rich in growth factors (PRGF) is an autologous platelet concentrate that locally releases growth factors and cytokines, supporting hemostasis and the regeneration of soft and bone tissues. It promotes wound healing, epithelialization, and may aid in the early resolution of chronic inflammatory lesions, such as periodontal pockets, making it useful in non-surgical periodontal therapy [107].
- EMDs: EMDs are used as adjunctive agents during periodontal therapy to promote the regeneration of both soft and hard tissues. They have demonstrated benefits such as reducing probing pocket depth (PPD) and increasing the clinical attachment level (CAL) [108].
- Blood-derived products: They are used in periodontology because they facilitate the regeneration of dental support tissues, promoting tooth retention by reconstructing the periodontium, collagen fibers, and alveolar bone [109].
- Engineered tissues, stem cells, and biomaterials: The main goal of this technique is to restore the function and structure of the tooth’s supporting tissues, which include the periodontal ligament, root cementum, and alveolar bone [110]. However, stem cell therapies and tissue engineering in the field of periodontology are still in their early stages.
- Antimicrobial agents: The local application of antimicrobial agents in the oral cavity in patients suffering from periodontitis offers a targeted and localized therapy. This approach achieves a drug concentration at the site of periodontal pockets that exceeds the minimum inhibitory concentration (MIC). This concentration remains high for up to several weeks, resulting in prolonged effectiveness over time and reducing the need for frequent administration [111]. CHX-based mouthwash, with concentrations between 0.1% and 0.2%, presents significant anti-plaque effects when used daily for a period of 2 weeks, even in the absence of mechanical cleaning. Furthermore, it appears to be a valid long-term complement to oral hygiene, if used at regular intervals of 4–6 weeks up to 6 months [112]. Among the antimicrobials used for the treatment of periodontitis, metronidazole in gel composition is indicated for its narrow spectrum of action against obligate anaerobes and for its fewer side effects [113], and tetracyclines for their MMP inhibitory capacity of plaque biofilm-induced degradation of periodontal structures. Among the tetracyclines, the favorite is doxycycline, which is safer and has superior pharmacokinetics [114].
- Anti-inflammatory agents: The use of NSAIDs in the treatment of periodontitis is still poorly defined, with effects on periodontal health that have not been fully clarified. Furthermore, since conventional periodontal therapies have been shown to be effective in resolving inflammation and stopping tissue destruction [115], the use of NSAIDs as an additional therapy is not necessary [72].

### 6.2. Future Perspectives

Recently, new products have emerged, including immunomodulatory mouthwashes that influence the immune response to oral microbes and nanoparticle-enriched mouthwashes for targeted antimicrobial action. Essential oils, such as red ginseng, aloe vera, propolis, fennel, thyme, ratanhia root, ginger, mallow, xylitol, bamboo charcoal, lemon, and curcumin, are increasingly incorporated into oral hygiene products like toothpaste, mouthwashes, and gels [116]. Dental probiotics also show potential in the management of gum disease. Probiotic mouthwashes contain live microorganisms, such as Lactobacillus, Bifidobacterium, Bacillus, and Saccharomyces, which can rebalance the oral microbiome, favor beneficial bacteria, and compete with pathogenic bacteria [117]. A recent technology is the use of nanoparticles in mouthwashes. These, measuring 1–100 nm, are used to improve the effectiveness of the product. In fact, chemicals bind to the surface of nanoparticles to protect the cargo from degradation, improve retention, and reduce the concentration of the drug required for therapeutic effects [116]. Regarding anti-inflammatory agents, research is currently focused on a new category, resolvins, as a replacement for NSAIDs. These compounds include derivatives of omega-3 fatty acids, docosahexaenoic acid, eicosapentaenoic acid, and lipoxins derived from arachidonic acid [118].

## 7. Conclusions

This review of the role of hyaluronic acid (HA) and re-epithelializing agents in periodontal regeneration has highlighted the potential of these biomaterials to improve clinical outcomes in the management of periodontal diseases. Hyaluronic acid, known for its anti-inflammatory, angiogenic, and osteoinductive properties, has been shown to promote cell migration, proliferation, and extracellular matrix synthesis, all essential functions for tissue healing. Its ability to modulate the inflammatory response by acting on receptors such as CD44 and RHAMM underscores its central role in coordinating wound healing and tissue regeneration. HA integrates into the extracellular matrix by forming associations with structural molecules such as collagen and fibrin, creating a microenvironment favorable to cell migration and proliferation. The molecular mechanisms by which HA influences regenerative processes include the modulation of cellular signaling pathways such as MAPK, PI3K/Akt, and NF-κB, which are crucial for the regulation of proliferation, matrix synthesis, and cell survival. The effects of HA on periodontal regeneration are particularly evident in its ability to enhance angiogenesis, facilitate collagen deposition, and modulate the behavior of fibroblasts and endothelial cells. This makes HA a promising candidate not only for improving therapeutic outcomes but also for reducing complications associated with conventional treatments. Epithelializing agents, including growth factors such as PDGF, EGF, and TGF-β, play a key role in stimulating cell proliferation and tissue repair. These agents promote extracellular matrix synthesis and enhance the regenerative capacity of periodontal tissues through the activation of intracellular signaling pathways. Their ability to interact with HA may create a synergistic effect that enhances tissue regeneration. Crosstalk between epithelial and connective tissue cells, mediated by HA and re-epithelializing agents, is of fundamental importance for successful periodontal regeneration. Interactions between fibroblasts and keratinocytes are modulated by growth factors and cytokines, which control cell proliferation and matrix synthesis. This bidirectional communication is crucial for maintaining tissue integrity and responding to inflammatory stimuli, facilitating the healing and regeneration of periodontal structures. In summary, hyaluronic acid and re-epithelializing agents represent powerful and versatile tools in periodontal regeneration, with the potential to improve clinical outcomes through the modulation of key cellular and molecular processes. Future research should focus on optimizing doses, delivery methods, and exploring potential combinations with other regenerative therapies to maximize the therapeutic potential of these agents. Progress in this field could open new avenues for personalized and more effective treatments for patients with periodontal disease, improving not only oral health but also the overall quality of life. The limitations of this study are mainly related to its design as a narrative review and the lack of a systematic approach. The present article does not focus on a specific outcome and lacks a qualitative analysis of the literature; it simply provides a descriptive analysis of the topic.

## Data Availability

The data are available from the corresponding author upon reasonable request.
